# Effect of the Hand-Omitted Tool Motion on mu Rhythm Suppression

**DOI:** 10.3389/fnhum.2016.00266

**Published:** 2016-06-02

**Authors:** Kazuo Isoda, Kana Sueyoshi, Yuki Ikeda, Yuki Nishimura, Ichiro Hisanaga, Stéphanie Orlic, Yeon-Kyu Kim, Shigekazu Higuchi

**Affiliations:** ^1^Graduate School of Integrated Frontier Science, Kyushu UniversityFukuoka, Japan; ^2^Advanced Business Center, Dai Nippon Printing Co., Ltd.Tokyo, Japan; ^3^Department of Multimedia, Cultural Bureau, Musée de LouvreParis, France; ^4^Faculty of Design, Department of Human Science, Kyushu UniversityFukuoka, Japan

**Keywords:** mirror neuron system, canonical neuron, tool use, electroencephalogram, human interface

## Abstract

In the present study, we investigated the effect of the image of hands on mu rhythm suppression invoked by the observation of a series of tool-based actions in a goal-directed activity. The participants were 11 university students. As a source of visual stimuli to be used in the test, a video animation of the porcelain making process for museums was used. In order to elucidate the effect of hand imagery, the image of hands was omitted from the original (“hand image included”) version of the animation to prepare another (“hand image omitted”) version. The present study has demonstrated that, an individual watching an instructive animation on the porcelain making process, the image of the porcelain maker’s hands can activate the mirror neuron system (MNS). In observations of “tool included” clips, even the “hand image omitted” clip induced significant mu rhythm suppression in the right central area. These results suggest that the visual observation of a tool-based action may be able to activate the MNS even in the absence of hand imagery.

## Introduction

“Mirror neurons” that discharge during both action done and the same action observed were first identified in the F5 area (ventral premotor cortex) in macaque monkeys (Gallese et al., 1996; Rizzolatti et al., 1996), which were subsequently found in the intraparietal sulcus, too (Fogassi et al., 2005). A number of experiments have suggested that this parieto-frontal cortical circuit in the observer of actions performed by other individuals encodes the goals and intentions of these actions (Rizzolatti and Craighero, 2004; Rizzolatti and Sinigaglia, 2010). In humans, functional magnetic resonance imaging (fMRI) studies have revealed the presence of mirror-like brain regions similar to those in monkeys. It has been proposed that the mirror neuron system (MNS) in humans is involved not only in the recognition of the goals and intentions of actions (Iacoboni, 2005), but also in imitation (Iacoboni, 2009; Molenberghs et al., 2009), empathy, facial expression recognition, and other social cognition functions (Preston and de Waal, 2002; Carr et al., 2003; Jabbi et al., 2007; Schraa-Tam et al., 2012). In the study using fMRI, disassociation for visual processing between ventral and dorsal was revealed during object and action recognition (Shmuelof and Zohary, 2005).

In addition to fMRI, electroencephalography (EEG) has been used for the measurement of the activity of the MNS in humans (Pineda, 2005). Specific alpha range of EEG have long been known which are present over the central area, corresponding to the primary motor and other areas, in an individual physically at rest and suppressible by not only her/his performing an action but also just observing the same action performed by another individual (Gastaut and Bert, 1954). Those EEG which have mirror-like characteristics and occur in the central area on the scalp of an individual physically at rest are also called “mu rhythm”. Since the first discovery of mirror neurons in 1996, a number of studies have revealed that mu rhythm are related to the MNS (Pineda, 2005). According to some of these studies, mu rhythm can be suppressed to varying degrees depending on the goal of an action to be observed (Muthukumaraswamy et al., 2004). Comparative EEG studies have also been reported using the test stimuli that are conventionally employed in fMRI studies (Perry and Bentin, 2009). Based on the study using both fMRI and EEG, mu rhythm are related to the activity of the primary motor cortex and the inferior parietal lobule (IPL) that reflects the firing of the MNS (Arnstein et al., 2011). These findings strongly suggest that, in humans, the level of mu rhythm can be an index of the activity of the MNS.

The evolution of the brain has endowed humans with the ability of tool-based actions. Although non-human animals can also perform tool-based actions, they cannot handle tools in as complex a way as humans. In humans and monkeys, visuomotor neural mechanisms that are involved in the visual observation and the handling of tools has been extensively investigated (Jeannerod et al., 1995; Järveläinen et al., 2004; Maravita and Iriki, 2004; Peeters et al., 2009; Costantini et al., 2011). However, the relation between the visual observation of the handling of tools and the activation of the MNS has been much less studied. A magnetoencephalography (MEG) study has reported that the primary motor area in an individual can be activated by just watching the motion of hands of another individual in tool-based actions (Järveläinen et al., 2004). fMRI studies in humans and monkeys also investigated the effect of watching a video featuring a tool-based action on the MNS (Peeters et al., 2009). As a result, a human-specific activity of the left IPL was identified, suggesting that this brain area is important in the tool-based actions in humans.

Similar mirror neurons are involved in brain activity induced by the visual observation of tools, which was first identified in monkeys (Murata et al., 1997). With the subject just focusing on the tool, these are neurons that respond, relying on the shape of the hand when it grabs the object and the pattern of movement. Rizzolatti et al. (2014) call them canonical neurons (Grèzes et al., 2003). According to a previous report in humans, the ventral premotor cortex, the posterior parietal lobe, and the precentral sulcus can be activated by the handling as well as the visual observation of a tool (Chao and Martin, 2000; Mecklinger et al., 2002; Grèzes et al., 2003). These activities are regarded as critical in the handling of tools. Furthermore, in tool action observations, mu rhythm suppression increased according to participant’s experience in the action (Cannon et al., 2014).

These previous reports are extremely intriguing as to the brain’s response to tool-based actions, but there has been hardly any research that distinguishes between tool and the presence of hands. By controlling the task, Shmuelof and Zohary (2005) succeeded in distinguishing reaction to an object and reaction to an action; yet, this is purely the observation of a reaction to an object as a subject matter. We will pay attention to the reaction to a tool that has been prepared as a medium to transmit the intended action to an object that is the subject. Research focusing on motion shows that mu rhythms are suppressed by biological motion but they will not be suppressed by random motion (Ulloa and Pineda, 2007). Indeed, there are reports that, even though mu rhythm suppression occurs when a person watches an image of a ball being thrown, just watching the ball in flight will not suppress the mu rhythm (Oberman et al., 2005). It is also unclear whether the MNS can be activated by watching the image of tools, hands, or both, because the test visual stimuli employed in previous studies included not only the image of a tool but also the image of hands that was handling the tool. If the MNS is activated by transmitting the intention of an action, even if the hand is omitted, it is probable that mu rhythm suppression will occur with just the movement of the tool.

In the present study, we investigated the effect of the image of hands on mu rhythm suppression invoked by the observation of a series of tool-based actions in a goal-directed activity.

## Materials and Methods

The participants were 13 healthy, right-handed university students (7 females and 6 males, 22.2 ± 1.3 years old) who normally do not engage in clay modeling work. The participants gave written informed consent to the present study only after they were provided with information on the test protocol. The study was approved by the Ethical Committee of Kyushu University.

As a source of visual stimuli to be used in the test, a video animation was chosen which a museum used to instruct its visitors on the porcelain making process (Louvre-DNP Museum Lab). This imagery consisted of processes such as clay kneading and wheel rotation (processes where tools are not used) as well as clay modeling using a kidney shaped profile and decoration (processes where tools are used), all performed by the hands of a porcelain maker.

In this study, first we looked to confirm MNS activity under circumstances not related to tools in order to confirm whether or not MNS activity via brain waves can be confirmed with the animation used for trials. Next, in accordance with the focus of this study, we looked to confirm the effect of just tool motion by comparing the presence/non-presence of hands under circumstances mediated by a tool. Note, that we conducted this experiment as a series of events.

**Figure 1:**
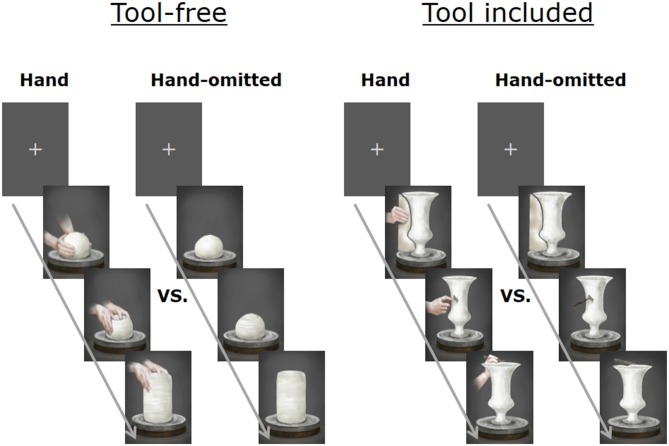
**Chapters on “tool-free” actions (e.g., clay kneading and wheel rotation: left panel) and chapters on “tool included” actions (e.g., clay shaping with a kidney: right panel).** The stimulus for baseline data was a still frame with a cross-hair at its center.

In order to elucidate the effect of the image of these hands, the image of hands were omitted from the original (“hand image included”) version of the animation to prepare another (“hand image omitted”) version. From each of these two versions, chapters on tool-free actions (e.g., clay kneading and wheel rotation: Figure 1, left panel) and chapters on tool-based actions (e.g., clay shaping with a kidney: Figure 1, right panel) were separately extracted and edited to make two shorter clips (“tool-free” and “tool included”). Each of the four shorter clips was presented repeatedly to each participant (70 s/clip). The control stimulus employed in the test was a still frame with a cross-hair at its center.

First, the participants were allowed to watch the whole movies (the “hand image included” version and the “hand image omitted” version). Then each of the four shorter clips was presented as a test stimulus. Before and after each shorter clip, the still frame control was presented (40 s). The “hand image included” stimulus and the “hand image omitted” stimulus were counter balanced between the participants for both the “tool-free” stimuli and the “tool included” stimuli.

EEG were measured in an electromagnetically shielded room (illuminance = 200 lx, temperature = 25°C, moisture = 50%). Each participant was seated on a chair and allowed to watch a series of the four clips and the still frame control on a liquid crystal display (19”), which was placed at 1.1 m from the chair. EEG were detected using a 64-channel EEG cap (Hydrocel GSN 64 ver.1.0, Electrical Geodesics, Inc.), with a low cut frequency of 0.3 Hz, a high cut frequency of 100 Hz, and a sampling rate of 250 Hz, which were A/D converted and recorded on a computer (PowerMac G5, Apple, Inc.) equipped with the Net Station 4.1.2 Software (Electrical Geodesics, Inc.).

The obtained data, except for those from the initial 10 s, were subjected to a frequency analysis (Fast Fourier Transform; FFT) at 1 epoch (4.091 s long) per 2 s. An average power value in the 10–12 Hz range, which was considered to well reflect the activity of the motor cortex (Pfurtscheller et al., 2000), was used as the mu power value. The calculated power values were normalized after logarithmic transformation, which were then analyzed using the EMSE Suite Data Editor 5.3 Release Candidate 3 Software (Source Signal Imaging, Inc.).

For each channel, we calculated mu rhythm suppression, i.e., the difference in the mu power value between each test stimulus and the control stimulus before/after the test stimulus. We set the motor area of the cerebral cortex region as the region of interest (ROI) to provide us with an indicator for confirming whether or not the MNS activity was increased by the movement of the tool with intention. The data from two of the participants contained missing values and outliers, and thus were excluded from the analysis.

**Figure 2:**
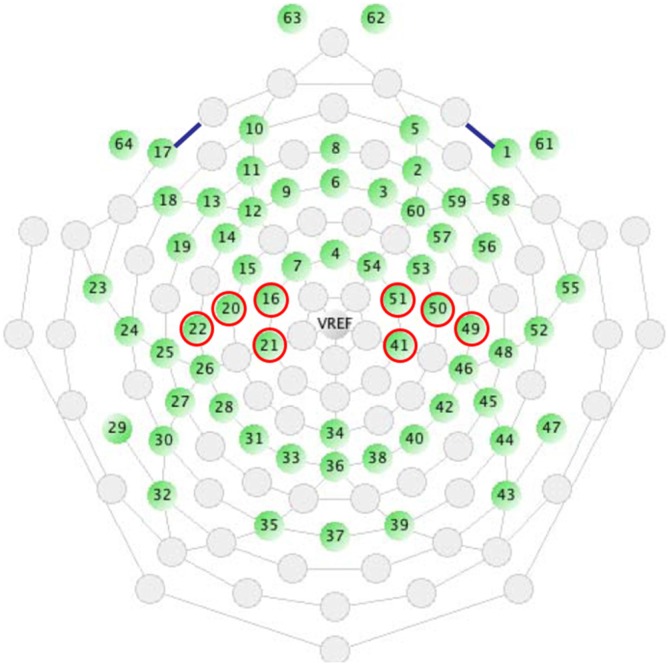
**Two regions of interest (ROIs) were defined and respective electrode sites were pooled: left central (16, 20, 21, 22) and right central (41, 49, 50, 51)**.

In order to enhance the reliability of the data, two ROIs were defined and respective electrode sites were pooled: left central (LC: electrodes 16, 20, 21, 22) and right central (RC: electrodes 41, 49, 50, 51; Figure 2). A paired *t*-test was performed to determine the significant mu suppression from the baseline data of the still frame. Next, a two-ways (hand image included/omitted × left/right hemisphere) repeated measurements of analysis of variance (rmANOVA) were conducted to determine the significance. Additionally, a paired *t*-test was used by using each individual electrode site of 64-channels and a three-dimensional topographic map (*t*-map) was generated.

## Results

### Analysis 1

**Figure 3:**
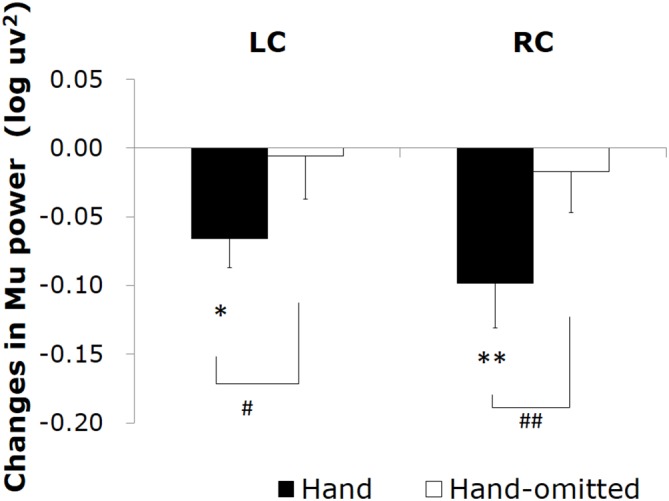
**Changes in the mu power value induced by watching the “tool-free” clips: “hand image included” (▪) and “hand image omitted” (□).** Asterisks (*p < 0.05, **p < 0.01) mean the significant mu rhythm suppression from baseline to observation of video clips. Sharps (^#^*p* < 0.05, ^##^*p* < 0.01) mean the significant differences between mu rhythm suppression by watching video clips “hand image included” and “hand image omitted”.

Changes in the mu power value induced by watching the “tool-free” clips are shown in Figure 3. Compared to the control stimulus by the still frame, a significant decrease in the mu power value was induced by the “hand image included” clip in the left central area (*t* = −3.13; *df* = 10; *p* = 0.011) and the right central area (*t* = −3.05; *df* = 10; *p* = 0.010). The “hand image omitted” clip did not induce any significant mu rhythm suppression.

**Figure 4:**
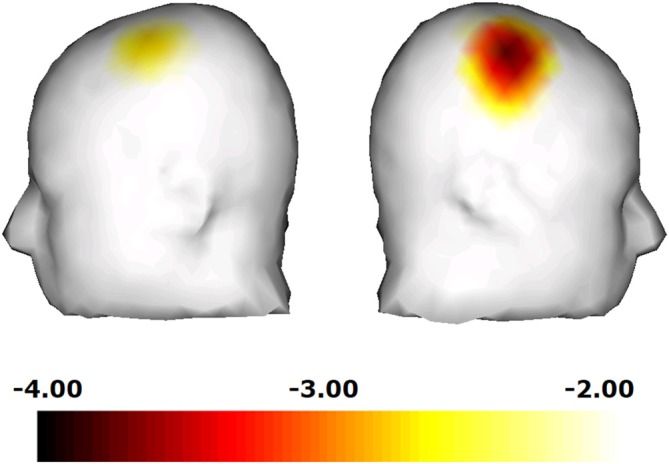
**Comparison for each electrode site, and plotted the results in a three-dimensional *t*-map, demonstrating the difference between the “hand image included” and “hand image omitted” clips specifically in the right central**.

We next compared the ability of the “hand image included” clip and that of the “hand image omitted” clip to induce mu rhythm suppression. As the results of two-way rmANOVA, although main effect of hand image was significant (*F* = 18.920; *df* = 1, 10; *p* = 0.001; *η^2^* = 0.654), main effect of hemisphere and interaction were not significant. It turned out that, compared to the “hand image omitted” clip, the “hand image included” clip induced a significant mu rhythm suppression in the right central area (*t* = −4.01; *df* = 10; *p* = 0.002) and the left central area (*t* = −2.57; *df* = 10; *p* = 0.028). We made a similar comparison for each electrode site, and plotted the results in a three-dimensional *t*-map (Figure 4), demonstrating the difference between the “hand image included” and “hand image omitted” clips specifically in the right and left central. No significant differences were found in other areas.

### Analysis 2

**Figure 5:**
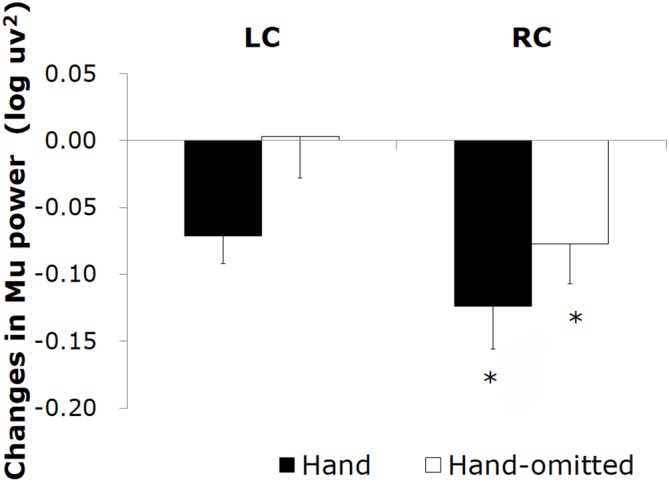
**Changes in the mu power value induced by watching the “tool included” clips: “hand image included” (▪) and “hand image omitted”(□)**.

**Figure 6:**
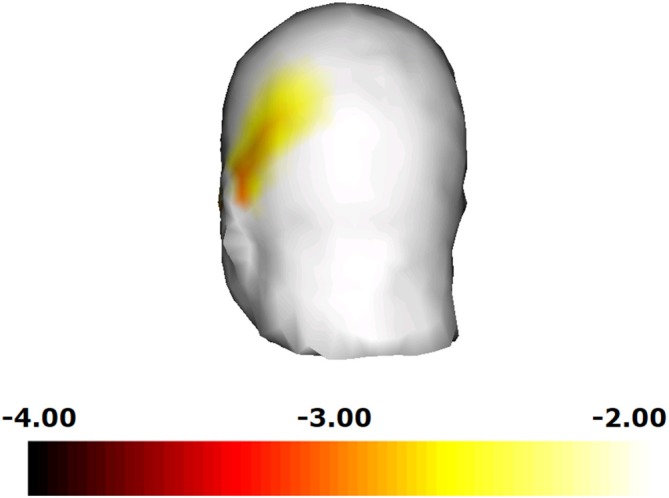
**Comparison for each electrode site, and plotted the results in a three-dimensional *t*-map.** We found a significant difference from the left parietal region to the left temporal region.

Changes in the mu power value induced by watching the “tool included” clips are shown in Figure 5. Compared to the still image control, a significant decrease in the mu power value was induced by the “hand image included” clip in the right central area (*t* = −2.26; *df* = 10; *p* = 0.035). In addition, the “hand image omitted” clip also induced a significant decrease in the mu power value in the right central area (*t* = −2.10; *df* = 10; *p* = 0.050). As the results of two-way rmANOVA, although main effect of hemisphere was significant (*F* = 7.299; *df* = 1, 10; *p* = 0.022; *η^2^* = 0.422), main effect of hand image and interaction were not significant. Mu suppression in right hemisphere was significant greater than that in left hemisphere. No significant differences were found for mu suppression between the “hand image included” and “hand image omitted” of the “tool included” clips. We made a comparison for each electrode site, and plotted the results by the corresponding three-dimensional *t*-map (Figure 6). In this analyze, we found also a significant difference from the left parietal region to the left temporal region (LP: electrodes 27, 30; Figure 2). No significant difference was found in other regions between these results.

## Discussion

“Tool-free” clips: comparison between “hand image included” and “hand image omitted”.

In analysis 1, we confirmed mu rhythm suppression only when hands were present, and even confirmed MNS activity with the animation video. In the observation of the “tool-free” clips by the participants, the “hand image included” clip induced a significant decrease in the mu power value in the central area (LC,RC), whereas the “hand image omitted” clip did not. These results are consistent with a previous observation that the visual observation of the motion of hands induced mu rhythm suppression in the central area (Muthukumaraswamy et al., 2004; Oberman et al., 2007; Perry and Bentin, 2009), indicating that, in the animation of the porcelain making process used in the present study, the image of hands activated the MNS in the motor cortex. Moreover, activity was seen in both the LC and RC hemispheres. This is conjectured to be hand movement representation of a contralateral preference that appears in the dorsal stream (Shmuelof and Zohary, 2005) while, in this study, another factor is considered to be that both hands were active in the presented hand movement.

In the “tool-free” clips, the “hand image included” clip also induced a decrease in the power value in the 10–12 Hz range in some areas other than the central area (LC,RC). This is thought to have happened because of the visual stimuli due to the clay images in the activity shared by presentations. The effect of the image of hands was investigated by comparing the effect of the “hand image included” clip and that of the “hand image omitted” clip on the power value in the 10–12 Hz range. (Figure 4) A significant difference was only present in the right central area, where the “hand image included” clip induced a significantly greater suppression of mu rhythm than the “hand image omitted” clip, which results in reaction to object (clay images) in the activity shared by presentations being offset, indicating a motor cortex-specific effect of the image of hands.

“Tool included” clips: comparison between “hand image included” and “hand image omitted”.

In analysis 2, regardless of whether or not the hand was present, we confirmed mu rhythm suppression in just the RC, and depending on the tool movement, we confirmed that MNS activity could be seen. In the observation of the “tool included” clips, even the “hand image omitted” clip induced a significant mu rhythm suppression in the right central area, resulting in activity being seen just in RC, which is thought to stem from the fact that the movement was nearly all presented on the left side of the screen. This too matches the contralateral tendency as described by Shmuelof and Zohary (2005), indicating that the motion of a tool can induce mu rhythm suppression in its observer even in the absence of the image of hands handling the tool. This is possibly because the motion of the tools (e.g., a kidney) may have compensated for the omitted image of the hands. According to previous studies in monkeys, as a result of watching a tool-based action, neurons in the parietal lobe can merge the tool into the hands handling the tool, leading to a cortical magnification (Hihara et al., 2006). In addition, it has been reported that the primary motor area can be activated not only by watching the motion of hands but also by just imagining the same motion (Pfurtscheller and Neuper, 1997; Pineda et al., 2000). Mu rhythm suppression induced by the “hand image omitted” clip in the central area might be attributable to an ability of the motion of a tool to evoke the image of the hands handling the tool in the brain.

Another possibility is the involvement of a brain activity that is induced by the visual observation of tools. It is known that the areas involved in the handling of tools can also be activated by just watching the tools (Chao and Martin, 2000; Grèzes et al., 2003). This characteristic activation of brain is seen in monkeys (Murata et al., 1997). Mecklinger et al. (2002) reported that the visual observation of a graspable object induces a stronger activity in the ventral premotor cortex than that of a non-graspable object. We suppose that such an activity induced by the observation of the motion of a tool may have induced mu rhythm suppression in the present study.

Similar to the “tool-free” stimuli, the “hand image included” clip in the “tool included” stimuli also induced a decrease in the power value in the 10–12 Hz range in many areas over the scalp. Therefore, we investigated the effect of the image of hands, by directly comparing the data from the “hand image included” and “hand image omitted” clips. As a result, the “hand image included” clip resulted in a significant mu rhythm suppression from the left parietal region to the left temporal region. From the two parameters compared, the only difference is the presence/non-presence of hands, so mu rhythm suppression in this case is considered to be the reaction variance regarding “hand movement”. As the presented “hand movement” is a right-hand one for manipulating a tool, a contralateral action that corresponds to the participant’s own hand movement is appearing. This matches the results showing a strong reaction to some of the images of a body shown in Downing et al. (2001). Yet again, viewed from a different perspective, according to a previous report based on both fMRI and EEG, the activity of the IPL is strongly related to mu rhythm suppression (Arnstein et al., 2011). In addition, it has been known that the observation of a tool-based action can activate the left IPL in the observer (Peeters et al., 2009). This activation is human-specific and not found in monkeys. In this study, the use of a tool is strongly related to the recognition of the difference in mu rhythm suppression between the “hand image included” and “hand image omitted” clips in the area corresponding to the IPL.

The present study has demonstrated that the visual observation of a tool-based action may be able to activate the MNS even in the absence of such an image of hands. This phenomenon may involve brain activities, which are known to fire in response to the visual observation of a tool. In the observation of the tool-based process, the image of hands induced mu rhythm suppression in the observer in the area corresponding to the IPL.

## Limitation

In this study, we adopted the commentary video animation of art works as a stimulus to evaluate museum information interfacing from the aspect of cerebral function. However, it is assumed that visitors to actual museums vary in characteristics, such as age, gender and profession. In previous research, it has been reported that the mu rhythm suppression is influenced by experiences (Cannon et al., 2014). Therefore, we need to take into consideration the experiences of participants when undertaking research. Indeed, although we validated the presence/non-presence of hands when presenting tool movement, we did not validate the presence/non-presence of a tool. To further stringently isolate influences, we should probably also carry out validation that can compare the presence/non-presence of the tool concerned.

## Author Contributions

Study conception and design: KI, KS, IH, SH. Acquisition of data: KI, KS, IH, SO, SH. Analysis and interpretation of data: KI, KS, Y-KK, YI, YN, SH. Drafting of manuscript: KI, KS, SH.

## Conflict of Interest Statement

The authors declare that the research was conducted in the absence of any commercial or financial relationships that could be construed as a potential conflict of interest. The Review Editor RJG and handling Editor JKS declared their shared affiliation, and the handling Editor states that the process nevertheless met the standards of a fair and objective review.
